# Layered Double Hydroxide Nanotransporter for Molecule Delivery to Intact Plant Cells

**DOI:** 10.1038/srep26738

**Published:** 2016-05-25

**Authors:** Wenlong Bao, Junya Wang, Qiang Wang, Dermot O’Hare, Yinglang Wan

**Affiliations:** 1 College of Biological Sciences and Biotechnology, Beijing Forestry University, 35 Qinghua East Road, Haidian District, Beijing 100083, P. R. China; 2 College of Environmental Science and Engineering, Beijing Forestry University, 35 Qinghua East Road, Haidian District, Beijing 100083, P. R. China; 3 Chemistry Research Laboratory, Department of Chemistry, University of Oxford, Mansfield Road, Oxford OX1 3TA, United Kingdom

## Abstract

Here we report a powerful method that facilitates the transport of biologically active materials across the cell wall barrier in plant cells. Positively charged delaminated layered double hydroxide lactate nanosheets (LDH-lactate-NS) with a 0.5‒2 nm thickness and 30‒60 nm diameter exhibit a high adsorptive capacity for negatively charged biomolecules, including fluorescent dyes such as tetramethyl rhodamine isothiocyanate (TRITC), fluorescein isothiocyanate isomer I(FITC) and DNA molecules, forming neutral LDH-nanosheet conjugates. These neutral conjugates can shuttle the bound fluorescent dye into the cytosol of intact plant cell very efficiently. Furthermore, typical inhibitors of endocytosis and low temperature incubation did not prevent LDH-lactate-NS internalization, suggesting that LDH-lactate-NS penetrated the plasma membrane via non-endocytic pathways, which will widen the applicability to a variety of plant cells. Moreover, the absence of unwanted side effects in our cytological studies, and the nuclear localization of ssDNA-FITC suggest that nano-LDHs have potential application as a novel gene carrier to plants.

Nanoparticle-based delivery technologies have unique advantages to transport exogenous molecules across the hydrophobic plasma membrane. They have played a central role in a wide variety of applications, including cell therapy, gene transformation, and the cellular delivery of molecular dyes^1^,^2^. Nanoparticles have many diverse potential applications, and a large number of nanoparticle groups have been developed including: viral carriers^3^, organic cationic compounds^4^, recombinant proteins^5^ and inorganic nanoparticles^6^. In all cases the selection of a suitable nanotransporter is crucial for successful application for specific target cells^7^.

Plant cells are characterized with their peripheral cell walls, which mainly consist of cellulose and pectin polysaccharides, which effectively protects plant cells from the penetration of foreign inorganic particles or deters pathogens attachment at the cell surface^8^,^9^,^10^. As a result, researchers have relatively few numbers of alternative approaches for the delivery of functional biomolecules into plant cells. For example, gene-transformation in plants is largely depended on the *Agrobacterium-*based methods, which is limited to a small plant group, including most dicots but only some monocots. The use of inorganic nanoparticles as nanotransporters has attracted great attention in recent years, as these materials are highly efficient and suitable for a wide range of cell types. However, the cell wall has on many occasions hindered the successful application of nano-sized delivery systems for plant cells. For instance, multi-walled carbon nanotubes (MWCNTs), mesoporous silica nanoparticles (MSN), TiO_2_ and CeO_2_ nanoparticles have all failed to penetrate the plant cell wall^11^,^12^,^13^,^14^. Fullerenes can internalize in plant cells but they exhibit high cytotoxicity by damaging the PM and cytoskeleton structures^15^. Polymer nanoparticles can successfully carry siRNA into the protoplast of BY-2 cells but not in cells with intact cell walls^16^. Thus, a suitable nanotransporter for living plant cells must have the following specific properties: (i) nanometer size to penetrate the cell wall, (ii) cause low damage to the PM, and (iii) exhibit a high absorbance capacity for the target molecule(s).

Layered double hydroxide (LDH) nanosheets appears to be emerging as an outstanding candidate nanomaterial in biology^17^,^18^. LDHs consist of positively charged hydroxide sheets with interlayers filled with charge-balancing anions and co-intercalated water^19^. The commonly used generalized formula of an LDH is [M^2+^_1−*x*_M^3+^_*x*_(OH)_2_][A^*n*–^]_*x/n*_·*z*H_2_O, where M^2+^ and M^3+^ are divalent and trivalent cations, respectively and A^*n*–^ is the interlayer anion^20^. Due to their unique structure and properties, LDHs have been widely applied as anion exchangers^21^, catalyst supports^22^, nanocomposites^23^,^24^,^25^, electroactive materials^26^, and gas adsorbent^27^,^28^. Several research groups have developed LDHs as efficient molecule transporter for the delivery of functional nucleotides and bio-active drugs^29^,^30^,^31^,^32^. These studies demonstrate that selected LDHs can be excellent transporters to living cells and show good biocompatibility and low cytotoxicity. Furthermore, the biomolecules may be protected within the layer galleries of LDH and only undergo pH-controlled release of their bioactive guests when the nanoplatelets located inside the cytosol^31^,^33^. LDHs can be exfoliated into single layers in many polar solvents, such as formamide, butanol and acrylates^19^,^20^. In 2005, researchers have successfully fabricated delaminated lactate-containing LDH 2D nanosheets in water, which have exceedingly high aspect ratios^19^,^20^,^34^. In the previous study, we found that [Mg_0.75_Al_0.25_(OH)_2_](C_3_H_6_O_3_)_0.25_ (Mg-Al-lactate LDH) loaded DNA molecules with high efficiency and facilitated delivery of DNA into human cells (293T) with low cytotoxicity^35^. These studies encouraged us to investigate the application of delaminated lactate-containing LDH nanosheets to deliver biologically active molecules to intact plant cells.

## Results

### Characterization of delaminated LDH and LDH-bioconjugates

**Figure 1:**
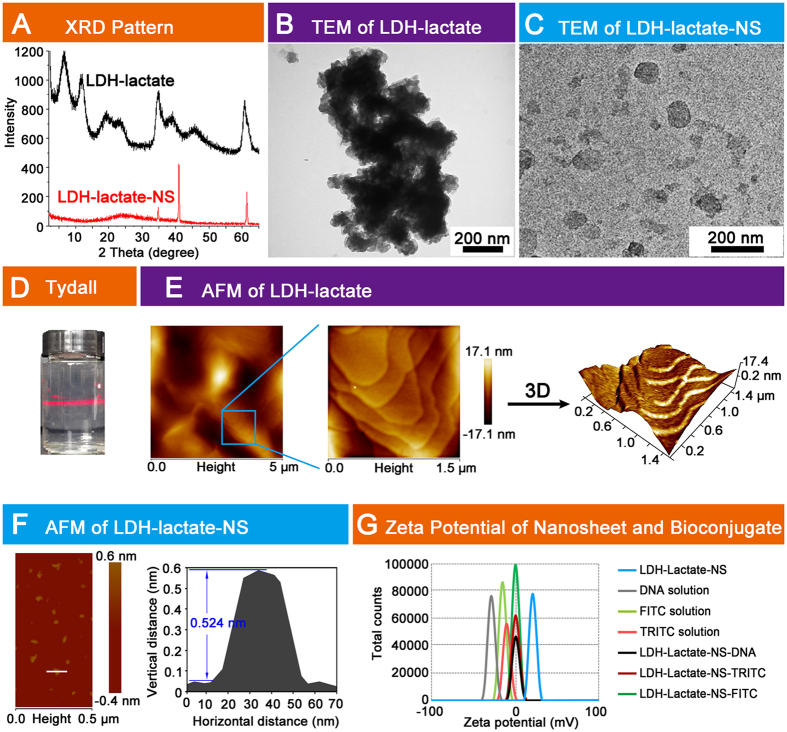
Characterization LDH-lactate and LDH-lactate-NS. (**A**) XRD patterns of LDH-lactate and LDH-lactate-NS; (**B**) TEM image of LDH-lactate; (**C**) TEM image of LDH-lactate-NS; (**D**) Tyndall effects of LDH-lactate-NS; (**E**) Left: overview AFM image of LDH-lactate. Middle: amplified field in the box. Right: three-dimensional image of amplified field; (**F**) Left: AFM image of delaminated LDH-lactate-NS, color bar indicates the vertical distance. Right: The vertical distance and horizontal distance of a LDH-lactate-NS marked with white line was measured and showed; (**G**) Zeta potential of DNA solution, LDH-lactate-NS, TRITC, FITC, LDH-lactate-NS-TRITC, LDH-lactate-NS-FITC, and LDH-lactate-NS-DNA suspension.

In this study, we synthesized bulk LDH-lactate by a coprecipitation method and then further delaminated the platelets in decarbonated water by following the previously described protocol^35^. The X-ray diffraction (XRD) patterns of a dried bulk Mg-Al-lactate sample is given in Fig. 1A (black curve), the XRD pattern exhibits the typical Bragg reflections which may be indexed as the *00l* reflections in addition to several broader and asymmetrically shaped *hk0* Bragg reflections at higher 2θ values. By intercalation of the lactate {CH_3_CH(OH)COO^−^} anions, the position of the *003* Bragg reflection (2θ = 6.49°) corresponds to d-spacing of 1.36 nm (Supplementary Table 1). After delamination, the characteristic XRD pattern of the LDH layer structure disappears. The absence of sharp basal plane 00*l* Bragg reflections indicates that there is no long range order in the platelet stacking direction after delamination (Fig. 1A, red curve). We have also used high resolution transmission electron microscopy (TEM) analysis to validate the morphology and ultrathin structure of the LDH-lactate and LDH-lactate-NS sample. The TEM images of LDH-lactate sample prior to delamination consisted of aggregated bulk crystallites (Fig. 1B). After delamination, high aspect ratio two-dimensional sheets with a translucent plate-like morphology were observed (Fig. 1C). Some faint sheets images were ascribed to weakly stacked structures, while the ultra-faint sheets were ascribed to single-layers. The lateral sizes of these nanosheets ranges from 30 to 60 nm. Furthermore, a clear Tyndall light scattering effect was observed for the colloidal suspension of LDH-lactate-NS, which indicates the presence of well-dispersed exfoliated nanosheets of the layered LDH-lactate (Fig. 1D). We also employed Atomic Force Microscope (AFM) to determine formation of the LDH-lactate and the LDH-lactate-NS. The AFM observation indicated that the LDH-lactate crystallites were multi-layered aggregates (Fig. 1E). In comparison, as shown in Fig. 1F, the apparent size of the nanoparticles is *ca*. 60 nm in lateral (*ab*) dimension which is in agreement with the FE-TEM data, the thickness of the film obtained from the corresponding height profiles is around 2 nm, which may be caused by the nanosheets layers assembled on the mica substrate. The smaller sheets generally showed a thickness of 0.5 nm, indicating that the LDH-lactate was delaminated into sheets with single molecular layer (Fig. 1F).

The delaminated LDH was conjugated with fluorescein isothiocyanate isomer I (FITC) and tetramethyl rhodamine isothiocyanate (TRITC) by electrostatic absorption to give LDH-lactate-NS-bioconjugates, namely LDH-lactate-NS-FITC and LDH-lactate-NS-TRITC. Zeta potential analysis showed that the exfoliated LDH-lactate-NS in slurries was positively charged (+27 mV) while the FITC, TRITC dispersions were both negatively charged, with the zeta potentials of −17.2 mV and −23.3 mV respectively. After the LDH-lactate-NS adsorption of FITC and TRITC, the zeta potential changes to a neutral voltage (Fig. 1G and Supplementary Table 2). We also tested the absorbance of LDH-lactate-NS to DNA molecules using zeta potential measurements. The initial salmon DNA mixture had a zeta potential of −30 mV, the final LDH-lactate-NS-DNA bioconjugates also had a neutral zeta potential (Fig. 1G and Supplementary Table 2).

### LDH-lactate ultrathin nanosheets as an effective biomolecular transporter for intact plant cells

**Figure 2:**
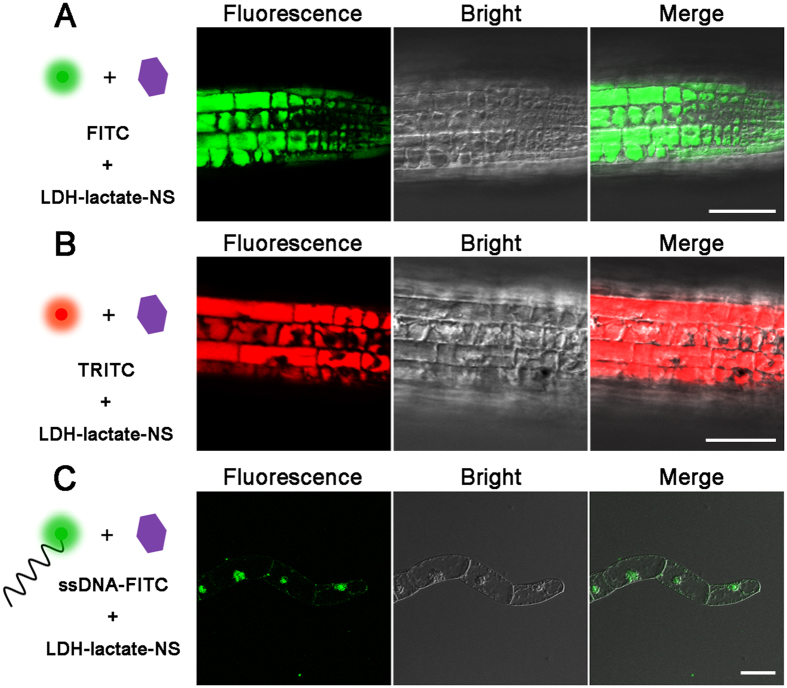
LDH-lactate-NS deliver biomolecules into plant cells. (**A**) Fluorescence of LDH-lactate-NS-FITC from cytosol of arabidopsis root cells; (**B**) LDH-lactate-NS-TRITC from cytosol of arabidopsis root cells; (**C**) Fluorescence of LDH-lactate-NS-ssDNA-FITC from cytosol of BY-2 cells. Scale bars = 50 μm.

In this study, we used the intact 5-day-old seedlings of *Arabidopsis thaliana* and Nicotianatobacum cv Bright Yellow 2 (BY-2) suspension cells as model systems to investigate the ability of LDH-lactate-NS as a molecular carrier for plant cells. The negatively charged fluorescent dye, FITC and TRITC, are membrane-impermeable (Supplementary Fig. 1). However, the neutral nano-platelet conjugates, namely the LDH-lactate-NS-FITC/LDH-lactate-NS-TRITC, are able to successfully shuttle the fluorescent dyes into the cytosols of the intact plant cells (Fig. 2A). Significant green (FITC) and red (TRITC) fluorescence were detected within the cytosol of epidermal cells from root apical region (Fig. 2A,B), as well as mesophyll and epidermal cells of leaves (Supplementary Fig. 2). Recording of the loading process in the BY-2 suspension cells indicated that the cytosolic fluorescence was increasing, when the cells were suspended within LDH-lactate-NS-FITC containing medium (Supplementary Video 1). After 10 minutes, the green fluorescence for FITC was obviously concentrated in the cytosol; the BY-2 cell showed stronger fluorescent intensity than the background fluorescence in the medium. With equal incubation time, higher concentration of LDH-lactate-NS-FITC in the culture medium resulted in stronger cytosolic fluorescence of both arabidopsis and BY-2 cells (Supplementary Fig. 3).

**Figure 3:**
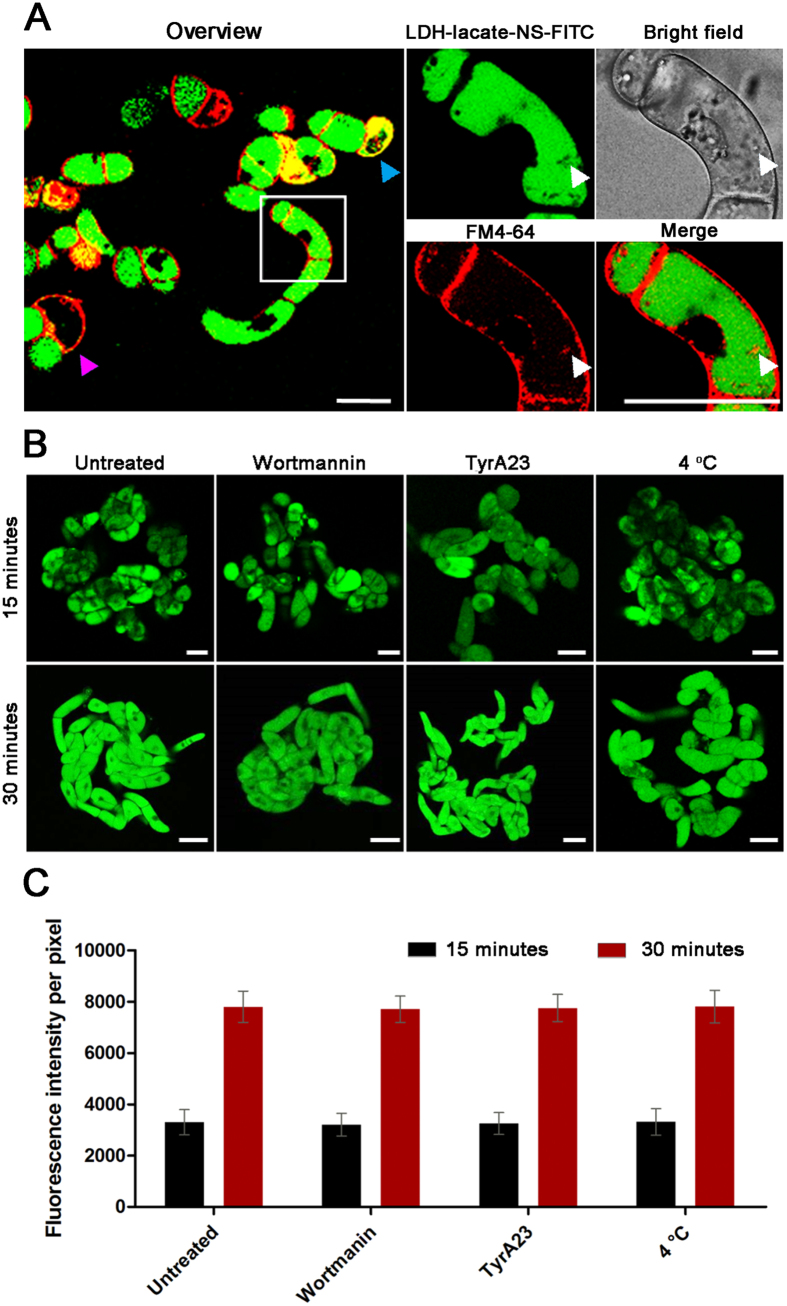
Investigation of the internalization pathways of LDH-lactate-NS in BY-2 cells. (**A**) Confocal images of BY-2 cells treated with LDH-lactate-NS-FITC followed with FM4-64 staining. The membrane integrity-destroyed BY-2 cell was marked with cyan triangle, and the magenta triangle represents the cell that non-internalization of LDH-lactate-NS-FITC. The white triangle indicate vesicle-like spots caused by endocytosis of FM4-64; (**B**) Wild type BY-2 cells (pretreated with wortmannin, tyrA23, 4 °C) incubated in 1/2 MS medium containing LDH-lactate-NS-TRITC; (**C**) The column chart present the mean fluorescence intensities that expressed as means ± standard deviation (SD, n = 150 cells in three independent replications). Scale bars = 50 μm.

Considering that suspensions of BY-2 cells have often been used as a model system for transient expression of target genes, we have investigated the penetration of DNA-conjugated LDH to intact BY-2 cells. Our findings clearly demonstrate that LDH-lactate-NS can deliver and specifically enrich the concentration of ssDNA-FITC in the nucleus of intact BY-2 cells (Fig. 2C), unlike the homogenous distribution of FITC molecules within the plant cell cytosols (Fig. 3A). Moreover, successful loading of LDH-lactate-NS-ssDNA-FITC into plant cellular nuclei requires a relatively long incubation time of around 60 minutes (Fig. 2C), while the ssDNA-FITC was impermeable to the PM of BY-2 cells.

### Internalization of LDH into living plant cells via non-endocytosis pathway

Previous studies have demonstrated that the internalization pathway for LDH-FITC into human MNNG/HOS is clathrin-mediated-endocytosis (CME)^36^. However, our observation on all kinds of plant cells revealed a homogeneous dispersion in the plant cell cytosol. It is logical to propose two potential mechanisms for such observations, one is the LDH-lactate-NS-FITC molecules were internalized via a CME pathway followed by pH-triggered release and subsequent free diffusion of FITC within the cytosol; the other is that the LDH-lactate-NS-FITC nanosheets can freely penetrate the PM. To investigate the relationship of LDH internalization between transport vesicles and plasma membrane (PM) structures, we sequentially incubated the BY-2 cells with LDH-lactate-NS-FITC for 15 min and N-(3-Triethylammoniopropyl)-4-(6-(4-(diethylamino)phenyl) hexatrienyl)pyridinium dibromide Pyridinium (FM 4-64) for 5 min. The CLSM image revealed that the green fluorescence was successfully loaded in most of the BY-2 cells. The red fluorescence from FM4-64 stained intact PM and a few endosomes in cytosols, suggesting that loading with LDH did not destroy the PM in this experimental condition (Fig. 3A). Moreover, the FITC fluorescence was absent from the FM4-64-labeled vesicle-like structures (Magnified images in Fig. 3A), indicating that the cytosolic FITC did not re-distribute into the endosomes.

To verify the internalization mechanism of the LDH into plant cells, we used two well-established inhibitors of endocytosis in plant cell biology, namely wortmannin and tyrophostin A23 (tyrA23)^37^,^38^. After 1 h pretreatment of BY-2 cells with wortmannin (33 μM) and tryA23 (33 μM) containing medium respectively, LDH-lactate-NS-FITC was added into the medium (with final concentration of 25 μg/mL). CLSM images indicated that these inhibitors did not prevent uptake of FITC fluorescence (Fig. 3B). Since low temperature can prevent endocytosis, we also repeated the LDH-lactate-NS-FITC uptake experiments at 4 °C. Again, clear green fluorescence was observed in the cytosol (Fig. 3B). To quantitatively determine the effects of these inhibitors, we measured the cytosolic fluorescence intensity of BY-2 cells after 15 and 30 min incubation under the treatments mentioned above. The mean values and standard deviations were shown as Fig. 3C. The statistical analysis allows us to conclude that neither the inhibitor treatment nor the low temperature inhibited the uptake of LDH-lactate-NS-FITC within the BY-2 cells.

**Figure 4:**
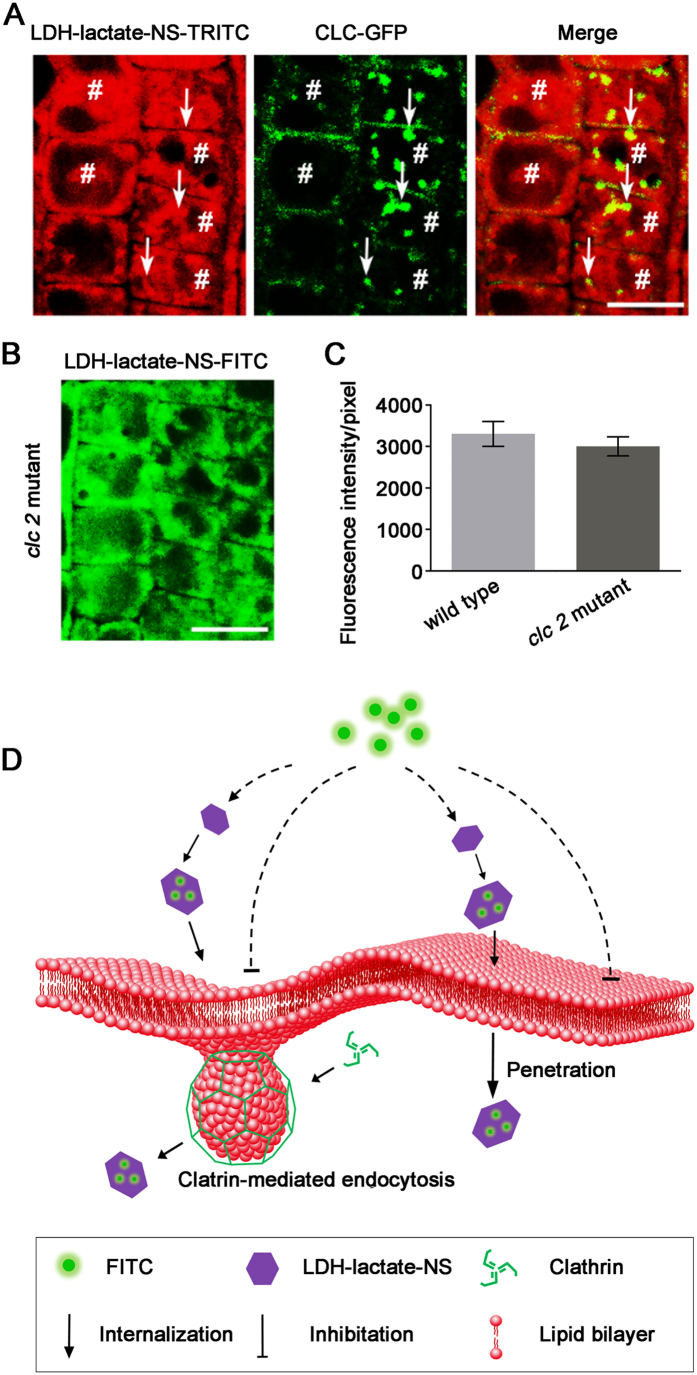
Cellular uptake of LDH-lactate-NS via free penetration mechanisms. (**A**) CLC-GFP transgenic arabidopsis incubated in 1/2 MS medium containing LDH-lactate-NS-TRITC. The arrow in each cell indicate the characteristic clathrin-mediated endocytosis spot, and the area of hash in each cell indicate uniform fluorescence from LDH-lactate-NS-TRITC. Scale bar = 10 μm; (**B**) Loading of LDH-lactate-NS-FITC into the root cells of clc 2arabidopsis mutant seedlings. Scale bar = 10 μm; (**C**) Fluorescence intensities from cytosol of the root cells of wild type arabidopsis seedlings andclc2mutant seedlings; (**D**) Schematic diagram of LDH-Lactate-NS internalization into intact plant cell.

We further used the transgenic arabidopsis lines to demonstrate the roles of CME in the uptake of LDH-lactate-NS-FITC in plant cells. When the LDH-lactate-NS-TRITC was loaded into arabidopsis cells with expression of clathrin light chain-GFP proteins (CLC-GFP), a few yellow spots indicated the partial co-localization of green (CLC-GFP) and red (LDH-lactate-NS-TRITC) fluorescence inside the cytosol (Fig. 4A). However, most of the TRITC fluorescence was still observed as a homogeneous dispersed feature without colocalization of CLC-GFP. Afterwards, we compared the loading rate of LDH-lactate-NS-FITC into the root cells of wild type arabidopsis seedlings and mutant line lacks the expression of *CLC2*, in which the CME is partially inhibited^39^. Statistical analysis on the fluorescence intensity inside the cytosol indicated that there was no significant difference between *clc2* and WT arabidopsis (Fig. 4B,C). These results revealed that CME is not crucial in internalization of LDH-lactate-NS into arabidopsis cells. Statistical analysis on the fluorescence intensity inside the cytosols indicated that there was no significant difference between *clc2* and WT arabidopsis (Fig. 4B,C). These results revealed that CME is not crucial for internalization of LDH-lactate-NS into arabidopsis cells.

## Discussion

In the cytological study of plant cells, the tough cell wall is not only a protective barrier to the outer environment, but also a barrier for the uptake of extracellular materials. The use of nano-materials for the delivery of active agents to plant cells is very limited. The plant cell wall was previously thought to prevent penetration of nanoparticles from reaching the cell membrane. However, the physiological properties of LDH suggest that it may be an ideal nano-delivery material for application in plants.

In this study we used positively charged Mg-Al-lactate LDH nanosheets with 0.5‒2 nm thickness and 30–60 nm lateral diameters to bind the negative charged fluorescent dyes (FITC, TRITC) and ssDNA (60-mer) by electrostatic absorption to form neutral nanosheet delivery system. Incubation of these nanosheets bioaggregates with living plant cells resulted in penetration between the cellulose fibers in cell walls, and internalization into the cytosol. The character of LDH nanosheets has provided two advantages over other nanoparticles: enhanced absorption surface area and an ultra-thin structural motif. As a result, a relatively low concentration (25 μg/mL) and a short incubation time (15 min) were sufficient for the successful incorporation of LDH-lactate-NS into arabidopsis root cells. These conditions were safe for the plant cells, according to the cytotoxicity analysis (Supplementary Figs 4 and 5).

LDH-lactate-NS can also be used as a nano-delivery system to suspended BY-2 cells. Time lapse video was used to show the dynamic LDH-lactate-NS-FITC internalization into the BY-2 cytosol. Intracellular FITC signals were detectable after 5 min incubation and exceeded the background environmental FITC signals after 15 min incubation. Importantly, the LDH-lactate-NS can easily deliver the ssDNA-FITC to the cell nucleus, which promises another great advantage of LDH-lactate-NS as a molecular transporter to cell-wall coated plant cells. In previous reports, single-walled carbon nanotubes (SWNTs) were successfully used as a nano-transporter to intact plant cells^40^. However, its application in plant cell biology was limited by the long incubation times (2 h), high cytotoxicity, and non-nuclear transport.

Understanding the internalization mechanism(s) for LDH nanosheets can provide clues for further improvement. Since the cellular internalization of LDHs was considered as CME in animal cells, we tested this possibility in living plant cells. Surprisingly, we cannot inhibit or even decrease the internalization rate of LDH-FITC within plant cells by inhibitor treatments and low temperatures. These experiments reveal that CME is not crucial in LDH-lactate-NS-FITC uptake, however, the partial colocalisation of LDH-lactate-NS-TRITC and CLC-GFP indicated that some LDH-lactate-NS-TRITC can be engulfed by the clathrin-coated endocytic vesicles. We further loaded the LDH-lactate-NS-FITC into the cytosol and labeled the PM with FM4-64 sequentially. These results indicated that the FM4-64-labeled endosomes were not colocalized with FITC signals. Since the latter incubation was in a medium without LDH-lactate-NS-FITC, this clearly demonstrates that the cytosolic FITC cannot be restored within membrane surrounded endosomes. Our data suggests that the FITC was not coupled with the LDH-lactate-NS as a membrane-permeable conjugate inside cytosols. This conclusion is also supported by the dynamic analysis of LDH-lactate-NS-FITC that the cellular FITC signals exceeded the environmental FITC signals, implying that the FITC were released from the neutral conjugates and lost the ability to release from plant cells.

The conclusion of LDH-internalization in plant cells is shown in Fig. 4D, the LDH-lactate-NS is both cell wall and membrane permeable to enable transport the biomolecules into plant cells. However, engulfing by clathrin coated vesicles did not exclude the LDH-lactate-NS bioconjugates and may slightly accelerate this process. Subsequently, the FITC/TRITC is released and dispersed within cytosols, while the ssDNA-FITC may localize and enrich within the plant cell nucleus.

In conclusion, our findings reveal that LDH nanosheets are an effective molecular delivery system to plant cells. The LDH nanosheets facilitate the delivery of fluorescent dyes into intact plant cells at low concentrations and very short incubation times. The absence of unwanted side effects in our cytological studies, and the nuclear localization of ssDNA-FITC makes LDH nanosheets a potential novel gene carrier candidate and suggest many possible uses in the emerging and promising field of plant nanobionic, that study the possibility of producing new biomaterial by the combination of nanomaterials with plant organelles.

## Materials and Methods

### Preparation of LDH nanosheet and nano-bioconjugates

Bulk Mg-Al-lactate LDH was synthesized using a coprecipitation method and delaminated in water into nano-scaled sheets. The experimental procedure was described in previous report^35^. The Mg-Al-lactate LDH wet slurry was added into the decarbonated water kept in N_2_ atmosphere. The final concentration of LDH-lactate dispersion was 1 g L^−1^, the whole system was stirred until no sediment was observed. The delaminated LDH-lactate is denoted as LDH-lactate-NS here. Tetramethylrhodamine isothiocyanate (TRITC) and fluorescein isothiocyanate isomer I (FITC) were purchased from Sigma-Aldrich (USA). To prepare the TRITC- and FITC-labeled nanosheets-conjugates, the prepared LDH-lactate-NS colloid (1 μg/μL) were mixed with FITC (1 μg/μL) and TRITC (1 μg/μL) aqueous solution with a volume ratio of 1:5. After centrifugation at 5000 rpm at room temperature for 5 min, the colloids were collected for further characterization. FITC coupled ssDNA was synthesized in TaKaRa Biotech. Co., Ltd (Dalian, P. R. China). The sequence of ssDNA-FITC was 5′-(FITC) GTTGGACTGTTCACAGAGATCGGCCCCATGTCCTGTTTCATCTCTCGTCACTCCATTCCC-3′. The ssDNA-FITC conjugates were made up to a stock solution in distill water (Mili-Q) with a final concentration of 1 mg/mL. This stock solution was diluted to final concentration of 0.2 μg/μL as work solution with MS medium used for cultivation of BY-2 cells. The delaminated LDH-lactate-NS colloid was added dropwise to ssDNA-FITC at a volume ratio of 1:1 followed by gentle mixing. The system was incubated for 15 min forming the LDH-lactate-NS-ssDNA-FITC conjugate. The zeta potentials of delaminated LDH-lactate-NS (1 g/L) and DNA (0.1 g/L) were measured using zeta potential analyzer (ZPA, Nanosizer Nano ZS, Malvern Instruments).

### Characterization of LDH nanosheet and nano-bioconjugates

The XRD patterns of synthesized LDH-lactate was recorded using a Shimadzu XRD-6000 instrument in reflection mode with Cu Kα radiation. The accelerating voltage was set at 40 kV with 30 mA current (λ = 1.542 Å, scan rate at 0.1°/s from 2θ = 5 to 65°. High resolution transmission electron microscopy (FE-TEM) images were obtained on a JEM-2010F, operating at 200 kV. Tapping mode atomic force microscopic (AFM) images were acquired under ambient conditions by directly casting sample dispersions onto freshly cleaved mica sheets using an Agilent Shimadazu SPM-9600 AFM system with Picoscan v5.3.3 software.

### Preparation of plant materials

*Nicotianatobacum* cv Bright Yellow 2 (BY-2) cells were cultured in media containing 0.43% [w/v] Murashige and Skoog (MS) salts (Sigma-Aldrich, USA), 1 mg/L thiamine, 0.2 mg/L 2,4-D, 100 mg/L Myo-inositol, 0.255 g/L KH_2_PO_4_, and 3% [w/v] sucrose (pH = 5.8). They were put in an orbital shaker at 130 rpm at 26 °C in the dark. After 3-4 days of cultivation, the cells were used for the following experiments. The seeds of *Arabidopsis thaliana* were sterilized for 10 min in 1.5% NaClO solution. Sterile arabidopsis seeds were then rinsed 5 times with sterile water and placed on MS growth-medium without or with LDH-lactate-NS (10, 25, 50, and 100 μg/mL) for germination and growth. Sterile petri dishes were used for all germination and growth experiments and placed into the growth chamber at 22 °C and 16/8 hours day/night rhythm.

### Uptake kinetics and binding assays

Prior to incubation with the LDH-bioconjugates, BY-2 cells were filtered with a cell strainer and resuspended in a sterile culture medium to obtain a well-dispersed cell suspension. 50 μLLDH-lactate-NS-bioconjugates (LDH-lactate-NS-FITC/TRITC or LDH-lactate-NS-ssDNA-FITC) was added to 450 μL cell suspension respectively, and the cells were placed on the shaker at 150 rpm and incubated in dark at 26 °C. For control experiments, cells were incubated with the same amount of FITC-labeled ssDNA or FITC only under the same conditions mentioned above. After incubation, the cells were washed three times to remove extra LDH bioconjugates or reagents, and then resuspended in the culture medium for immediate confocal imaging.

### Cell treatment

0.45 μL wortmanin (Sigma-Aldrich, USA) and tyrphostin A23 (tyrA23, Santa Cruz, USA) stock solution (33 mM in DMSO) was added to 450 μL of the cell suspension and incubated with the cells for 30 min. Then 50 μL LDH-lactate-NS-bioconjugates was added to the cell medium as mentioned above. After incubation at 26 °C for 15 min, the cells were rinsed several times with a standard growth medium and then imaged to calculate the quantitative subcellular fluorescence intensity per pixel. For the 4 °C treatment, the experiment was achieved by incubation of the cells on ice.

### Confocal microscopy imaging

10 μL of well-dispersed cell was dropped onto a glass slide after incubation with LDH-lactate-NS-bioconjugates (LDH-lactate-NS-FITC, LDH-lactate-NS-TRITC and LDH-lactate-NS-ssDNA-FITC), the fluorescence was captured with an inverted laser scanning microscopy (Leica SP8, Germany). The FITC was excited with 488 nm laser and the emission fluorescence was collected at wavelengths between 500‒550 nm. The TRITC was excited with 545 nm laser and the emission fluorescence was collected as wavelengths between 570–630 nm. The chlorophyll was excited by 488 nm laser and the emission auto-fluorescence was collected at wavelengths between 700‒750 nm. Two objectives were used in this study: 10x objective (NA = 0.3 Leica, Germany) and 100x objective (NA = 1.35, oil immersion, Leica, Germany). The intensity was calculated by averaging the fluorescence intensities of cells (about 150 cells) from the images of each sample using Image J software.

### Cell viability assay

5 μL Propidium Iodide (PI) (30 μM) was added to 50 μL suspension of cells incubated with MS growth medium containing different concentration of LDH-lactate-NS (10, 25, 50, 100, 300, and 500 μg/mL). The cells were incubated for 15 min in the dark at 26 °C, washed 3 times with growth medium, and then imaged by inverted laser scanning microscopy (Leica SP8, Germany). The specimen was excited at 535 nm for PI and fluorescence signals of 570 to 670 nm were collected for PI in the red fluorescence channel. Untreated cell groups were considered as the positive control, and the relative cell viability was calculated by the formula as follows: Relative cell viability (%) = TC/UC × 100, where TC indicate living cells percentage from treated cell groups and UC represent living cells percentage from untreated cell groups.

## Additional Information

**How to cite this article**: Bao, W. *et al*. Layered Double Hydroxide Nanotransporter for Molecule Delivery to Intact Plant Cells. *Sci. Rep.* **6**, 26738; doi: 10.1038/srep26738 (2016).

## Supplementary Material

Supplementary Information

Supplementary Information
