# Supplementary material for: Layered Double Hydroxide Nanotransporter for Molecule Delivery to Intact Plant Cells
Source: Sci Rep. 2016 May 25;6:26738. doi: 10.1038/srep26738 (PMC4879670; doi:10.1038/srep26738)
Supplement: Supplementary Information [file srep26738-s1.doc]

Layered Double Hydroxide Nanotransporter for Molecule Delivery to Intact Plant Cells

Wenlong Bao^a,#^,Junya Wang^b,#^, Qiang Wang^b^,*, Dermot O’Hare^c^, Yinglang Wan^a^,*

^a^ College of Biological Sciences and Biotechnology, Beijing Forestry University, 35 Qinghua East Road, Haidian District, Beijing 100083, P. R. China

^b^ College of Environmental Science and Engineering, Beijing Forestry University, 35 Qinghua East Road, Haidian District, Beijing 100083, P. R. China

^c^ Chemistry Research Laboratory, Department of Chemistry, University of Oxford, Mansfield Road, Oxford OX1 3TA, United Kingdom

^#^These two authors contributed equally to this work

*Corresponding authors:

Professor Yinglang Wan

E-mail: [ylwan@bjfu.edu.cn](mailto:ylwan@bjfu.edu.cn)

Tel: +86 18612523332

Professor Qiang Wang,

E-mail: [qiangwang@bjfu.edu.cn](mailto:qiangwang@bjfu.edu.cn); [qiang.wang.ox@gmail.com](mailto:qiang.wang.ox@gmail.com)

Tel: +86 13699130626

**Supplementary information**

Supplementary Tables

Supplementary Table 1. The 2 d-spacing of each peak of LDH-lactate XRD pattern

| 2 Theta (degree) | 6.49 | 11.4 | 19.3 | 23.6 | 34.9 | 38.4 | 45.8 | 60.7 |
| --- | --- | --- | --- | --- | --- | --- | --- | --- |
| d-spacing  (nm) | 1.36 | 0.74 | 0.46 | 0.38 | 0.26 | 0.23 | 0.2 | 0.15 |

Supplementary Table 2. Raw zeta potential value of each aqueous solution

| Aqueous solution | FITC | TRITC | | DNA | LDH-lactate-NS |
| --- | --- | --- | --- | --- | --- |
| Zeta potential (mV) | -17.2 | -23.3 | | -30.0 | +27.0 |
| Aqueous solution | LDH-lactate-NS-FITC | | LDH-lactate-NS-TRITC | LDH-lactate-NS-DNA |  |
| Zeta potential (mV) | 0 | 0 | | 0 |  |

**Supplementary Figures**


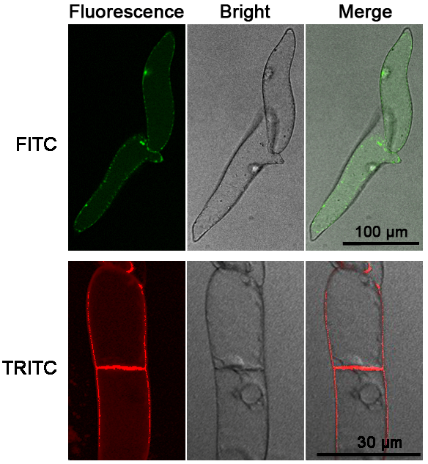


**Supplementary Fig. 1.** FITC and TRITC is membrane-impermeable dye to plant cells. BY-2 cells were incubated in FITC and TRITC containing medium for 1 hour in room temperature. Scale bars are indicated in the images.


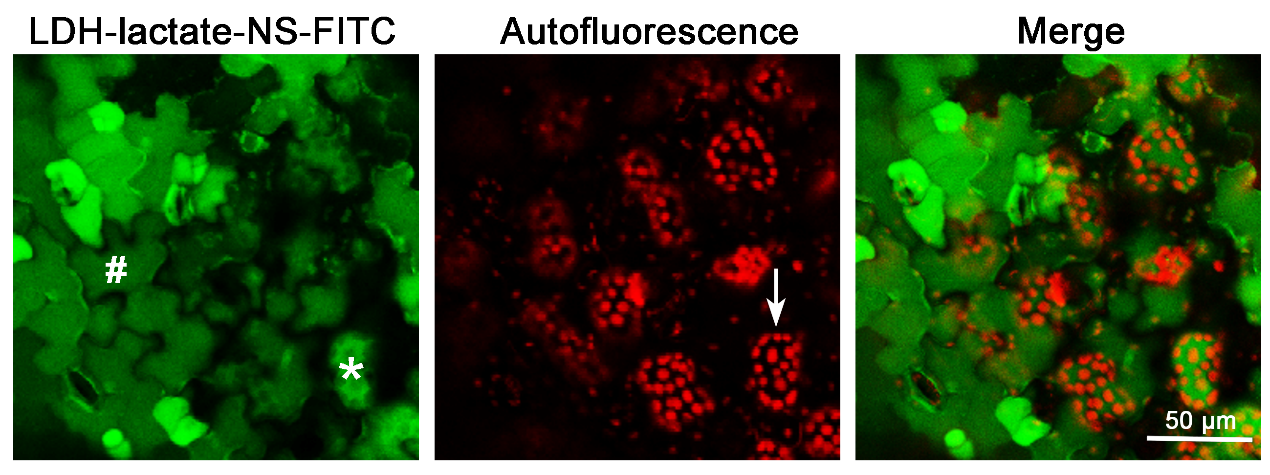


**Supplementary Fig.** **2.** LDH-Lactate-NS delivers FITC into mesophyll and epidermal cells of leaves. The epidermal cell of leaves was marked with the hash, and the star represents mesophyll cell inside leaves. The arrow presents chloroplast inside mesophyll cells. Scale bar = 30 µm.


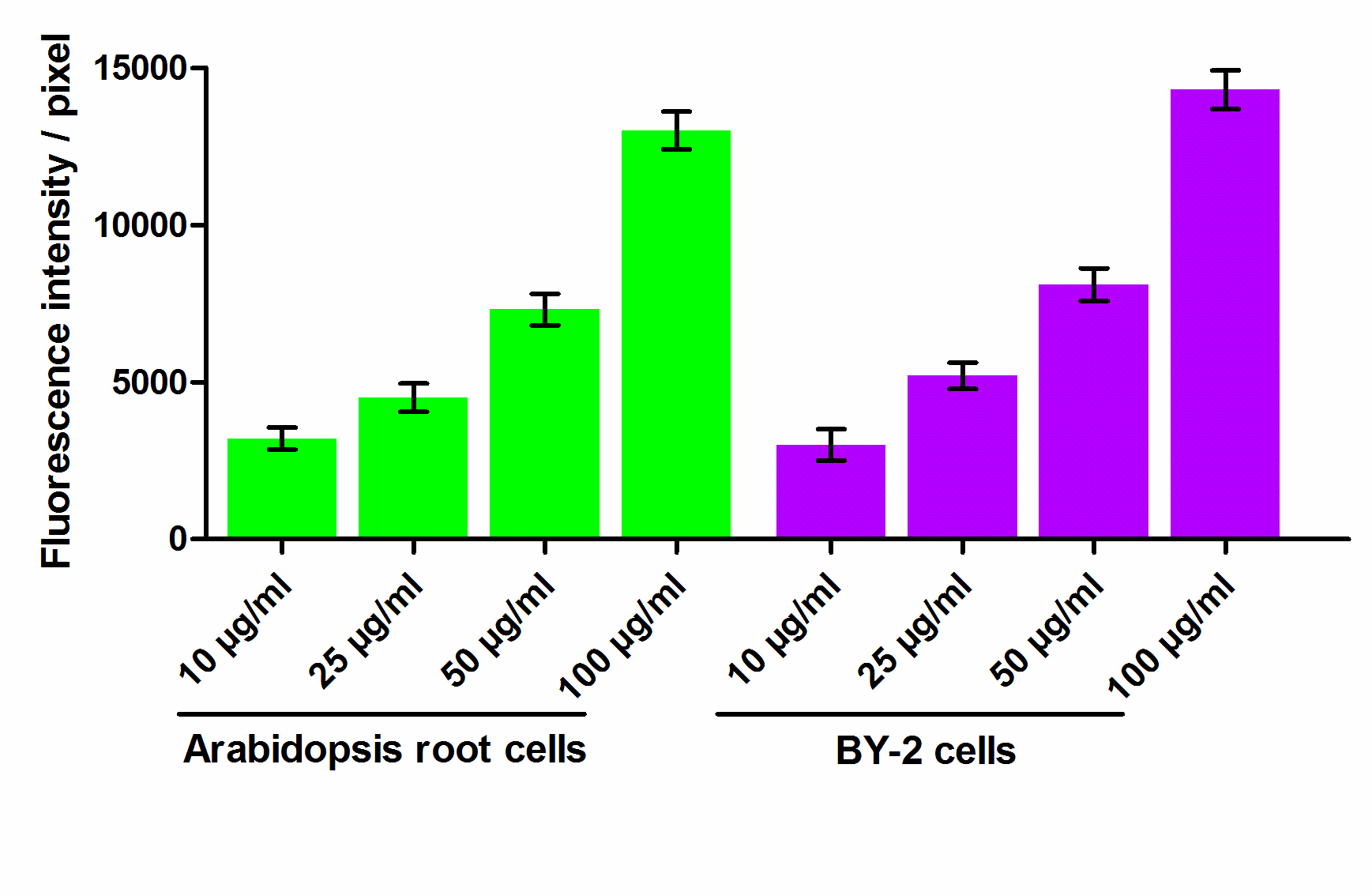


**Supplementary Fig. 3.** Statistical analysis of fluorescence intensity from cytosols of arabidopsis root epidermal cells and BY-2 cells. Green columns represent the fluorescence intensity from arabidopsis root epidermal cells incubated in 1/2 MS medium containing different concentration of LDH-lactate-NS-FITC(final concentration of 10, 25, 50, and 100 μg/ml), and the purple columns present the fluorescence intensity from BY-2 cells under the same experimental condition with arabidopsis root cells. Results are expressed as means ± standard deviation (SD).


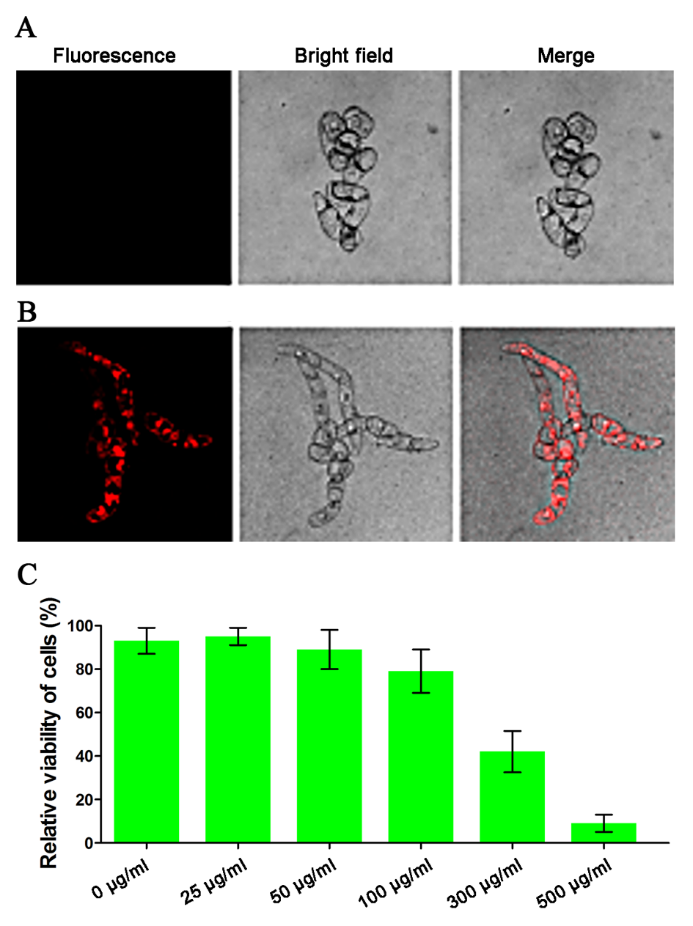


**Supplementary Fig. 4.** Cell viability was examined by staining the cells with Propidium Iodide (PI) dye.(A) Healthy BY-2 cells after PI staining;(B) dead BY-2 cells after PI staining;(C) Relative viability of BY-2 cells by counting 100 cells after pretreatment of 1 hour in different concentration of LDH-lactate-NS. Means ± SD were calculated from three independent experiments.


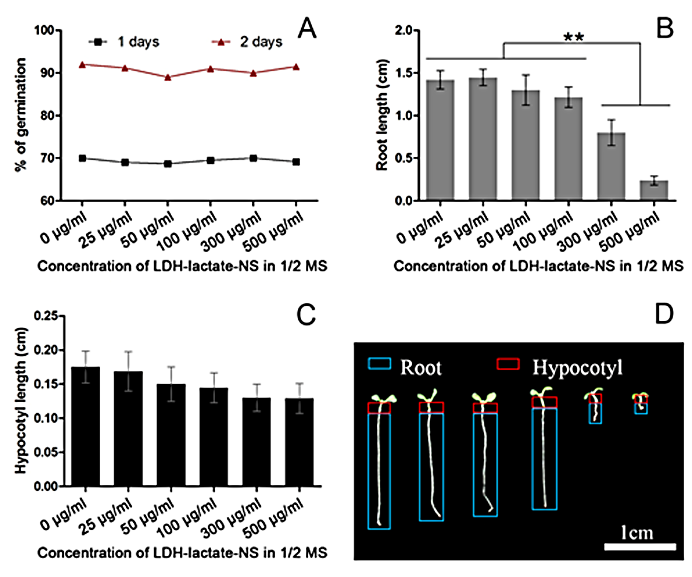


**Supplementary Fig. 5.** Effects of LDH-Lactate-NS on (A) seed germination, (B) root length, and (C) hypocotyl length of *Arabidopsis thaliana*. (D) Root and hypocotyl part of the seedlings treated with 1/2 MS medium containing different concentration of LDH-lactate-NS (from left to right 0, 25, 50, 100, 300, and 500 μg/ml) was marked with red and blue frame respectively. Results are expressed as means ± standard deviation of the mean value (SD). 50 individual seedlings were measured in each treatment (n=50).

**Supplementary Video 1.** A time lapse video recorded the uptake of LDH-lactate-NS-FITC in the liquid medium contains 25 μg/mL LDH-lactate-NS-FITC conjugates. 40 images were taken in 20 minutes, and built up a 5 seconds video here.
